# Role of Inosine–Uracil Base Pairs in the Canonical RNA Duplexes

**DOI:** 10.3390/genes9070324

**Published:** 2018-06-28

**Authors:** Naďa Špačková, Kamila Réblová

**Affiliations:** 1 Department of Condensed Matter Physics, Faculty of Science, Masaryk University, Kotlářská 2, 611 37 Brno, Czech Republic; spackova@monoceros.physics.muni.cz; 2 CEITEC—Central European Institute of Technology, Masaryk University, Kamenice 5, 625 00 Brno, Czech Republic

**Keywords:** adenosine to inosine editing, dsRNA, molecular dynamics simulations, I-U base pairs

## Abstract

Adenosine to inosine (A–I) editing is the most common modification of double-stranded RNA (dsRNA). This change is mediated by adenosine deaminases acting on RNA (ADARs) enzymes with a preference of U>A>C>G for 5′ neighbor and G>C=A>U or G>C>U=A for 3′ neighbor. A–I editing occurs most frequently in the non-coding regions containing repetitive elements such as ALUs. It leads to disruption of RNA duplex structure, which prevents induction of innate immune response. We employed standard and biased molecular dynamics (MD) simulations to analyze the behavior of RNA duplexes with single and tandem inosine–uracil (I–U) base pairs in different sequence context. Our analysis showed that the I–U pairs induce changes in base pair and base pair step parameters and have different dynamics when compared with standard canonical base pairs. In particular, the first I–U pair from tandem I–U/I–U systems exhibited increased dynamics depending on its neighboring 5′ base. We discovered that UII sequence, which is frequently edited, has lower flexibility compared with other sequences (AII, GII, CII), hence it only modestly disrupts dsRNA. This might indicate that the UAA motifs in ALUs do not have to be sufficiently effective in preventing immune signaling.

## 1. Introduction

RNA editing represents an alteration of naturally occurring nucleobases in transcribed RNA. The most common RNA modification is the replacement of exocyclic amino group in adenine for carbonyl oxygen at the C6 position leading to inosine (A–I change). This process is mediated by the adenosine deaminases acting on RNA (ADAR) enzyme which acts on double-stranded RNA (dsRNAs) [1,2,3]. In mammals, ADAR has two active members, ADAR1 and ADAR2 [4,5,6]. Algorithm and web-based programs were developed to determine the sequence nearest neighbor preference for editing A–U pairs in dsRNA. For 5′ neighbor, the preference was U>A>C>G, and for the 3′ end, it was G>C=A>U for human ADAR1 and G>C>U=A for human ADAR2 [7]. Experimental coefficients describing editing frequencies are in Table S1. The low preference for guanine at the 5′ end, but high preference at the 3′ end, was explained based on the experimental structure of ADAR2 bound to dsRNA [8]. It was shown that the amino group of guanine at the 5′ end creates a clash with the protein backbone at residue Gly489, while the amino group of guanine at the 3′ end is important for proper hydrogen bonding with Ser486. Inosine, when compared with adenine, has different bonding pattern, hence it is read as guanine in coding regions, which results in the protein diversity [2,9,10]. Interestingly, in mammals, most editing occurs in the non-coding regions of transcripts containing repetitive elements such as inverted ALUs, which often form dsRNA [11,12]. A–I editing of these elements prevents induction of innate immune response and preliminary apoptosis, which is normally triggered by dsRNA generated during viral replication [13,14]. The inhibition probably results from specific binding of dsRNA with I–U pairs to cytosolic sensors such as MDA-5 or RIG-I [15,16]. In particular, dsRNA that has a disrupted structure due to I–U pairs [17] impedes protein binding and/or immune signaling [18]. Thus, ADAR activity responsible for A–I modification also controls innate immune response and facilitates the discrimination of self and non-self dsRNA [15].

The three-dimensional (3D) structure of RNA duplex with I–U pairs was determined only in one study, in which I–U formed tandem I–U/I–U base pair steps [19]. It was observed that I–U pairs affect the local structure and stacking, but do not induce distortion of the global duplex structure. The I–U pair, similar to the guanine–uracil (G–U) pair, represents a wobble pair, which is near-isosteric to canonical A–U or G–C pairs [20]. The stability of single I–U pairs was compared with G–U and A–U pairs in the melting experiments [21]. On average, the internal I–U pairs were less stable than A–U and even less stable than G–U pairs. It was suggested that the G–U pair is more stable than the isosteric I–U either because of better stacking in the duplex or because of stabilization by a water bridge between the amino group of guanine and 2′-hydroxyl of uracil. Similarly, duplexes with tandem I–U/I–U base pairs were less stable in melting experiments than duplexes with standard canonical base pairs (determined in one sequence context) [17].

In this study, we employed molecular dynamics (MD) simulations to investigate the role of single and tandem I–U base pairs in dsRNA duplexes at positions that were found to be deaminated by ADAR1 [22]. This computational technique provides insight into structural dynamics of molecules on a nanosecond (ns) to microsecond (μs) time scale, and was instrumental in previous studies focused on RNA and DNA duplex flexibility [23,24,25,26,27,28,29]. We performed a detailed structural analysis of 500 ns long standard simulations. Besides, an adaptive biasing method (ABF) [30] enhanced by the multiple walkers approach (MWA) [31], was employed to get a better view on the local flexibility and behavior of I–U pairs in different sequence contexts. Using this approach, we can overcome high energetic barriers that separate the thermodynamic states of interest, which is not feasible with standard MD. In contrast to the traditional umbrella sampling method, where a reaction coordinate is divided into discrete values sampled in series of windows using restraints, which may affect the sampled statistical distribution, the ABF method is unrestrained. An adaptive bias compensates the free energy barriers, so the sampling is reasonably uniform, and the free energy landscape is accurately described [32]. With the MWA, we can further improve sampling by running multiple simulations, walkers, which exchange information about the accumulated mean force in regular intervals. If the walkers are independent, the sampling (e.g., quality of the calculated free energy) is improved linearly with the number of walkers.

## 2. Materials and Methods 

**Figure 1:**
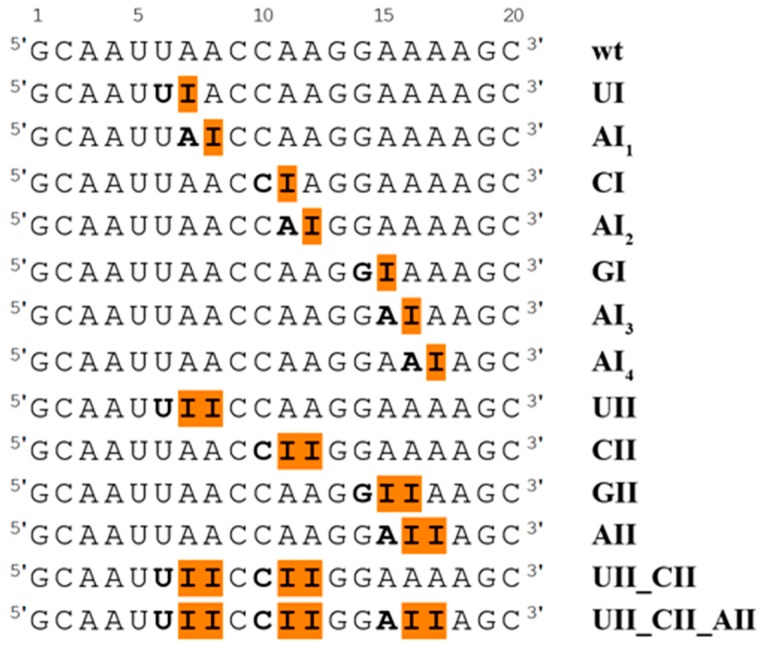
Summary of all duplexes simulated in this study presented as a sequence of the first strand. Inosines are highlighted by orange boxes, and the sequence used for simulation naming is in bold.

We studied RNA duplex with sequence 5′-GCAAUUA_7_A_8_CCA_11_A_12_GGAA_16_A_17_AGC-3′, where adenines at positions 7, 8, 11, 12, 16, and 17 were replaced by inosines, according to the experimental study [22]. We also studied the system with inosine at position 15, where the adjacent base at the 5′ end is guanine (this was not observed experimentally). Unmodified (wt) dsRNA duplex and 13 dsRNA duplexes with single or tandem I–U pairs in different sequence context were prepared for MD simulations. A survey of all studied systems is presented in Figure 1.

**Figure 2:**
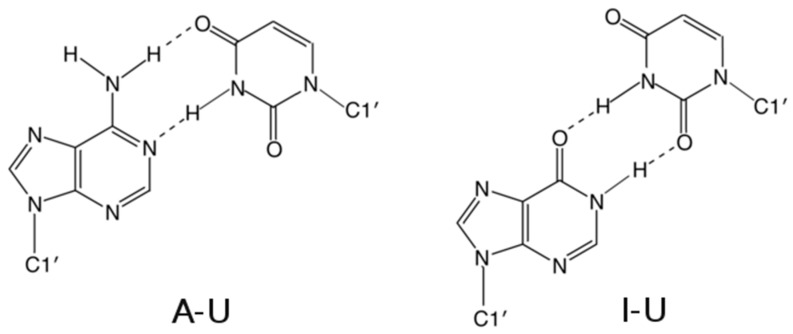
Schematic representation of adenosine–uracil (A–U) and inosine–uracil (I–U) base pairs. Hydrogen bonds are shown as dashed lines.

The RNA duplexes were built by the NAB module of AMBER 14 [33] as a right-handed A-RNA using a parmbsc0χOL3 force field [34]. The force field parameters for inosine were taken from the library of modified residues [35] and adjusted according to a used force field. Replacement of adenine by inosine in the wild type structure is related to an unfavorable geometry of the I–U base pair, which is characterized by strong hindrance between O6(I) and O4(U), as well as between N1–H1(I) and N3–H3(U) atoms (Figure 2). This steric and electrostatic clash was eliminated by a 2000-step restrained minimization in vacuo. Harmonic restraints of 20 kcal/mol·Å^2^ were applied to distances N1(I)–O2(U) and O6(I)–N3(U) corresponding to hydrogen bonds in the I–U base pair. The whole molecule, except for the I–U pair, was fixed by position restraints of 20 kcal/mol·Å^2^. The final structure with the correct geometry of the I–U base pairs was solvated by a periodic octahedral box of TIP3P water molecules extending 10 Å away from the solute and neutralized with sodium counterions [36] with Xleap module of AMBER. Equilibration and production setup used in this study exactly corresponds to the standard protocol applied by the ABC consortium to the large-scale MD studies of nucleic acids [37,38]. The standard length of MD simulations was 500 ns. Several simulations were extended to the length of 1 µs.

Interaction energies were calculated with Anal module of AMBER. In these calculations, we considered only bases in a given mutual geometry while the rest of the system was neglected. The atomic charges were not modified after dissection. We evaluated the interaction energy of H-bonding E_H-bond_ (between two bases forming a base pair) and the interaction energy of stacking E_stack_ (between two base pairs). The interaction energy is defined as the energy difference between an assembly (base pair or base pair step) and its parts (bases or base pairs) separated into infinity. Calculations were performed in vacuo and included only the van der Waals and electrostatic terms. These interaction energies provide only an approximate insight into the stability, because solvent screening is not included.

Parameters of base pairs, base pair steps, and total bend were obtained with Curves [39]. Local bend of base pair steps was calculated as described by Sherer [40], based on tilt and roll parameters obtained with X3DNA [41] with the following formula.

(1) $$\mathrm{bend}=\sqrt{{\mathrm{roll}}^{2}+{\mathrm{tilt}}^{2}}$$

The molecular mechanics, Poisson–Boltzmann combined with surface area (MM-PBSA) method was used for calculation of the energy of formation of RNA duplexes. The calculations were performed on snapshots directly from the simulation. We calculated the energies as a difference between the energy of the duplex and energies of individual strands:

(2) $$\Delta E_{\mathrm{formation}}=E_{\mathrm{duplex}}-\left(E_{\mathrm{strand}1}+E_{\mathrm{strand}2}\right)$$

Because of slow entropy convergence, the entropic term was not included in these calculations as it causes the largest fluctuations in the overall free energy. We aimed to compare the relative energies among the RNA duplexes. Nevertheless, to capture and compare flexibility of RNA duplexes with a different number of I–U pairs and various sequence context, a vibrational entropy contribution was calculated based on a coordinate covariance matrix with Ptraj module of AMBER in time windows of 100–200 ns, 100–300 ns, 100–400 ns, and 100–500 ns for phosphorous atoms. The obtained values were fitted with linear regression to derive an estimate of *S*_∞_ that is independent of the length of the MD simulation, see example in Figure S1.

The stability of I–U pairs was also investigated with biased MD simulations. Five different restart files from the unrestrained production MD simulations were used as the starting coordinates for subsequent parallel ABF [30] calculations accelerated by the MWA [31]. This ensured independence of the starting configurations for MWA walkers. All ABF/MWA simulations were performed in the modified PMEMD program from AMBER connected with PMFLib [42]. The I–U base pairs were perturbed via the change of shear parameter sampled in the interval from −5 Å to +5 Å, which was discretized into 100 bins. Since the base pairing outside the interval is not well defined, the shear was kept within the interval by wall restraints with the force constant of 40 kcal/mol·Å^2^. Based on our tests, the free energy profiles were converged with 1 × 10^6^ points sampled along the defined range, which is usually reached with a 300 ns long simulation (the simulation consisted of at least five parallel 60 ns long simulations). If some regions were not sampled sufficiently, the ABF simulation was extended. We analyzed the I_7_–U_34_ pair in UI and UII, the I_11_–U_30_ pair in CI and CII, the I_15_–U_26_ pair in GI and GII, and the I_16_–U_25_ pair in AI_1_ and AII systems. We also investigated the standard A–U pair in different sequence contexts in the wild-type (wt) system, that is, we probed A_7_–U_34_, A_11_–U_30_, A_15_–U_26_, and A_16_–U_25_ in separate ABF simulations. The obtained dependence of the free energy on the shear provides an estimate of the energy cost when bases forming I–U pair are shifted from the original geometry into major or minor grooves. The starting position of A–U pair inside the duplex shows shear around 0 Å, while the starting position of I–U pair in the duplex is modestly shifted towards major groove, so its shear is about −2.4 Å. In addition, we perturbed the I_7_–U_34_ pair in UII, the I_11_–U_30_ pair in CII, the I_15_–U_26_ pair in GII, and the I_16_–U_25_ pair in AII tandem systems via base pair parameter opening sampled in the range from −80° to 80°.

The MD trajectories were processed with the Ptraj module of AMBER and visualized with VMD program [43]. For classification of the substates, the H-bond distance cutoff was set to 3.4 Å to also include minor fluctuations of these intermolecular distances. According to the AMBER manual and Wood et al. [44], the H-bond angle cutoff was set to 135°.

## 3. Results

### 3.1. Description of Base Pairing

**Figure 3:**
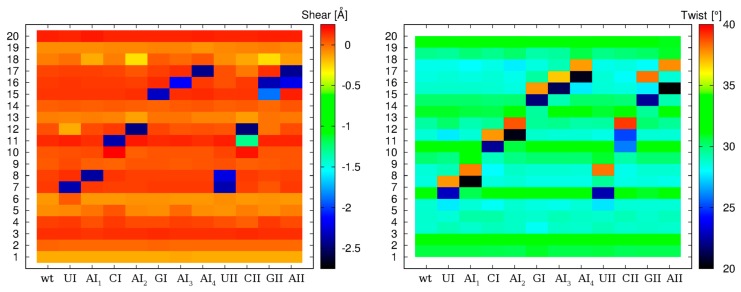
Averaged values of shear (**left**) and twist (**right**) calculated for twelve selected systems. Shear was calculated for each base pair, the twist was calculated for each base pair step (residue numbers are on the *y*-axis). Data were averaged along the whole trajectory.

The basic geometry of the I–U base pair (Figure 2) is stabilized by two hydrogen bonds formed between N1(I)–O2(U) and O6(I)–N3(U) atoms. In comparison to the canonical A–U base pair, uracil participating in I–U base pairing does not use its O4 atom (which is the H-bond acceptor in A–U base pair), but rather its O2 atom. This fact is reflected in changes of several parameters, mainly of shear and twist (Figure 3). While the canonical A–U base pair adopts zero shear, the shear of the I–U base pairs is approximately −2.4 Å. The average value of the twist is 29.5° for base pair steps not containing the I–U base pairs. If the canonical base pair is followed by the I–U base pair, the twist of this base pair step decreases to 21.5°. On the other hand, when the I–U base pair is followed by the canonical base pair, the twist increases to 38° and fully compensates for the decrease of the twist in the previous step.

Thus, the global twist is not influenced by the presence of the I–U base pair. These changes of the twist are observed for both the single and the tandem I–U base pairs. The twist for the I–U/I–U base pair step adopts the standard value mentioned above. We also detected an increase of local bend for tandem I–U/I–U steps of about 2–3 Å with respect to corresponding A–U/A–U steps in wt systems (Table S2). These changes are rarely seen for single I–U base pairs.

In our study, we focused on both the isolated single I–U pairs and two consecutive I–U base pairs forming the I–U/I–U base pair step.

### 3.2. Systems with the Single I–U Base Pair

The single I–U base pair was studied in several sequential contexts, namely in UIA, AIC, CIA, AIG, GIA, and AIA sequences, corresponding to UI, AI_1_, CI, AI_2_, GI, and AI_3_ or AI_4_ systems, respectively (Figure 1). The behavior of the I–U base pair is not influenced by its neighboring 5′ and 3′ base pairs. The I–U base pair predominantly adopts the initial geometry with an occasional opening into the major, and less frequently into the minor, grooves corresponding to C- and B-geometry described in the Section 3.3 (distributions of possible geometries for selected single I–U systems are available in Figure S2).

### 3.3. Systems with the Tandem I–U/I–U Base Pairs

**Figure 4:**
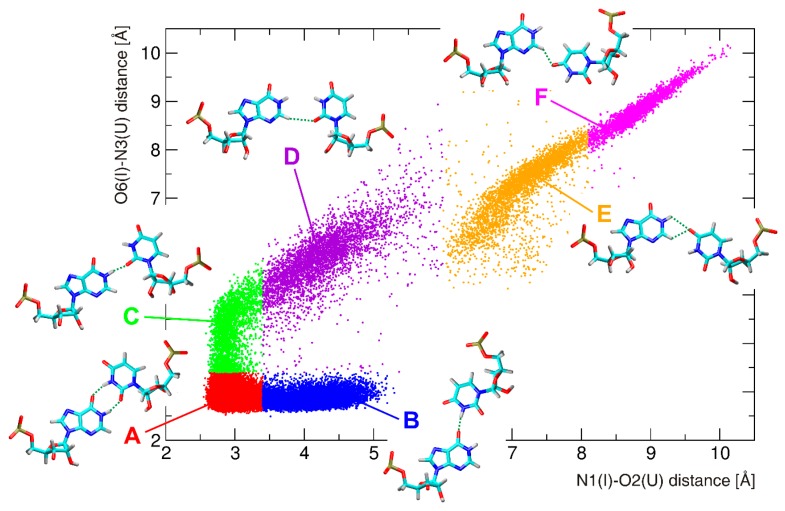
Distribution of various geometries of the first I–U base pair observed in the CII sequence context as a result of correlation between N1(I)–O2(U) and O6(I)–N3(U) H-bond distances. Substate populations are roughly distinguished by a different color and completed by representative geometries of I–U base pairs. Hydrogen bonds are shown as dashed lines.

The tandem I–U base pairs were studied in the four sequence contexts, namely, UIIC, CIIG, GIIA, and AIIA sequences, corresponding to UII, CII, GII, and AII systems (Figure 1). An analysis of RNA properties was carried out on equilibrated structures as demonstrated by root-mean-square deviation (RMSd) plots in Figure S3. The second I–U base pair of the tandem exhibits the same behavior as the single I–U base pair described above, while the first I–U base pair is much more variable and its geometry is strongly dependent on the neighboring 5′ base pair. A deep H-bond analysis revealed six possible I–U base pair geometries. All of these geometries are shown in Figure 4, which reflects their distribution in the CII system (distributions for other simulated systems are available in Figure S4). The populations of these substates within studied systems are presented in Table 1.

The base pair geometry named A represents the basic geometry characterized by the formation of two H-bonds, N1(I)–O2(U) and O6(I)–N3(U). Although it represents the most populated geometry in all four systems, the population of this substate substantially varies in simulated systems. While the A-geometry predominates with 90% in the UII system, it is populated only by 56% in the CII system. The UII system represents the most rigid system, which is predominantly locked in the basic geometry.

Two geometries, namely B and C, represent a variation of the A-geometry that is characterized by preservation of one H-bond and disruption of the other one leading to the opening of the base pair to the minor or major grooves. In case of the B-geometry, the O6(I)–N3(U) H-bond is preserved, while the N1(I)–O2(U) H-bond is disrupted, and the base pair opens to the minor groove. In case of the C-geometry, a preserved H-bond is N1(I)–O2(U), O6(I)–N3(U) is disrupted, and the base pair opens to the major groove. The B-geometry is mostly populated (21%) in the CII sequence, while the C-geometry is mostly populated (12%) in the AII. The opening of the I–U base pair is only marginally populated in the UII and GII systems.

Other geometries (named D, E, and F) are generally less populated, and they are characterized by disruption of both former H-bonds, which are replaced by new ones. The D-geometry is stabilized by only one H-bond formed between C2(I) and O2(U). Because of an unfavorable orientation of hydrogenated nitrogens, the base pair geometry is not planar. The D-geometry is a result of a shear toward the minor groove and is characterized by almost zero value of this base pair parameter. Geometries named E and F are characterized by the participation of O4(U) atom on stabilizing H-bonds. O4(U) atom can be an H-bond acceptor for hydrogens of N1(I) and C2(I). A single N1(I)–O4(U) H-bond is not frequently observed, more often we can observe a bifurcated N1/C2(I)–O4(U) H-bond, which is typical for the E-geometry. This geometry is non-negligibly populated only in the CII and GII systems. The F-geometry is characterized by a single C2(I)–O4(U) H-bond, but this geometry is significantly populated only in the CII system. Two views on the A-geometry (in the UII system) and the F-geometry (in the CII system), as well as an overlay of the A- and F-geometries (in the CII system), are available in Figure S5.

Our MD simulations show that the stability of the tandem system is determined by the neighboring 5′ base pair. The most stable system contains uracil at the 5′ end. This system is locked in the basic A-geometry for most of the simulation time. On the other hand, the presence of cytosine at the 5′ end introduces pronounced flexibility into the system. The tendency of flexibility can be expressed as C>A=G>U.

Systems with multiple tandem I–U/I–U base pairs (i.e., UII_CII and UII_CII_AII) exhibit very similar behavior of the individual tandem base pairs with almost the same distribution of various geometries. 

### 3.4. Energetic Profiles of the I–U Pair Based on Adaptive Biasing Method Calculations and Correlation with Standard Simulations

**Figure 5:**
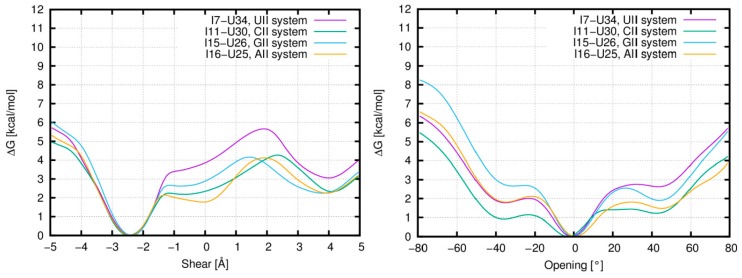
Free energy profiles of shear (**left**) and opening (**right**) for the first I–U base pair in the I–U/I–U tandem systems based on adaptive biasing method (ABF) simulations. ΔG: Gibbs free energy variation.

**Figure 6:**
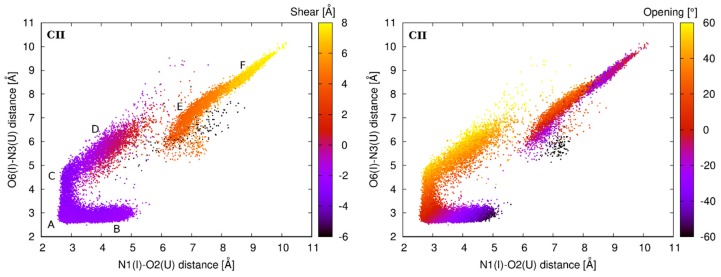
Distribution of the shear (**left**) and opening (**right**) parameters of the first I–U pair in CII system based on the unbiased molecular dynamics (MD) simulation. Color scale represents the shear/opening.

The energetic profile of the shear for the first I–U pair from tandem I–U/I–U systems shows three minima (Figure 5 left): the global minimum is identical for all studied systems and is observed for the shear around −2.4 Å. This value corresponds to the basic base pair A-geometry and also to the B- and C-geometries detected in standard simulations (Figure 6 left). Around the shear value of 0 Å, there is a shallow minimum corresponding to the D-geometry (the D-geometry is mostly populated in the CII and AII systems, while it is rarely observed in the UII system (Table 1)). Another minimum is observed around 4 Å, which represents the E-geometry. This geometry can be detected in all tandem systems except for the UII, and this fact correlates with the higher value of ΔG for this minimum (Figure 5 left).

The energetic profile of the opening (Figure 5 right) also contains three minima. However, their relation to the I–U base pair geometries is not straightforward, as in case of the shear. The global minimum around 0° represents mainly the A-geometry with the partial participation of the E- and F-geometries (Figure 6 right). Another minimum is found around the value of −35°. This represents the opening into the minor groove, which is a characteristic feature of the B-geometry. It is not surprising that the lowest energy is observed for the CII system where the B-substate is mostly populated. A minimum is also observed around 45°, which mainly corresponds to the D-geometry. Again, this minimum is not preferred by the UII system, which is in agreement with our MD data.

### 3.5. Interaction Energy Analysis

Substitution of the A–U base pair for the I–U base pair is connected with energetic changes. H-bonding interaction energy of the I–U base pair is around −11 kcal/mol, which is 1 kcal/mol lower than E_H-bond_ of the A–U pair, of which energy is −10 kcal/mol. In the case of the single I­–U base pair, the stacking between I–U and its 5′ neighbor is destabilized by 1–2 kcal/mol, while the stacking between the same I–U pair and its 3′ neighbor is not substantially affected. Thus, a modest improvement of the H-bonding energy and slight destabilization of the stacking energy results in no significant impact on the overall single I–U base pair stability. This agrees with the behavior of single I–U pair in standard simulations.

In the case of the tandem systems, the stacking between the first I–U base pair and its neighboring 5′ base pair is influenced by the I–U geometry, and is described in the following paragraph. The stacking between the second I–U and its neighboring 3′ base pair is similar to that in the single I–U systems. Additionally, the I–U/I–U tandem represents the weakest stacking interaction with the E_stack_ around −2.5 kcal/mol, compared with A–U/A–U stacking, which exhibits E_stack_ around −7 kcal/mol.

### 3.6. The Flexibility of the First I–U Base Pair and Its Impact on the Interaction Energy

**Figure 7:**
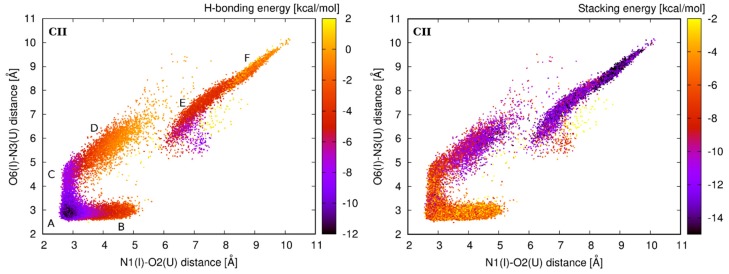
Distribution of the interaction energies in CII system. **Left**: E_H-bond_ between I_11_ and U_30_ residues of the first I–U base pair. **Right**: E_stack_ between the I_11_–U_30_ base pair and its 5′ base pair neighbor C_10_-G_31_. Color scale represents the interaction energy.

Geometrical alterations of the first I–U base pair are also connected with changes in interaction energies. Interaction energy of the I–U base pair is affected by disruption of original H-bonds and formation of alternative ones. Distribution of H-bond interaction energy E_H-bond_ for the CII system is shown on the left in Figure 7. The most stable configuration of the I–U base pair (E_H-bond_ around −11 kcal/mol) is the initial A-geometry with two interconnecting N1(I)–O2(U) and O6(I)–N3(U) H-bonds. Other substates are less stable because of only one H-bond contact.

Fluctuations of the first I–U base pair in tandem I–U/I–U also affect stacking interaction energies (E_stack_) of corresponding base pair steps. Considering the I–U/I–U stacking initial A-geometry has E_stack_ only around −2.5 kcal/mol, while the alternative geometries, mainly D and F, adopt more stable stacking arrangement (E_stack_ around −6 kcal/mol).

The stacking interaction between the first I–U base pair and its 5′ base pair neighbor in the initial A-geometry varies between −8 kcal/mol (for UII) and −6 kcal/mol (for CII). Except for the CII system, alternative geometries have very similar or less favorable stacking energies. In the case of the CII system (see the distribution of E_stack_ in Figure 7 right), E_stack_ for the alternative D-, E-, and F-geometries is substantially lower and varies between −10 kcal/mol (for D-geometry) and −14 kcal/mol (for F-geometry).

**Figure 8:**
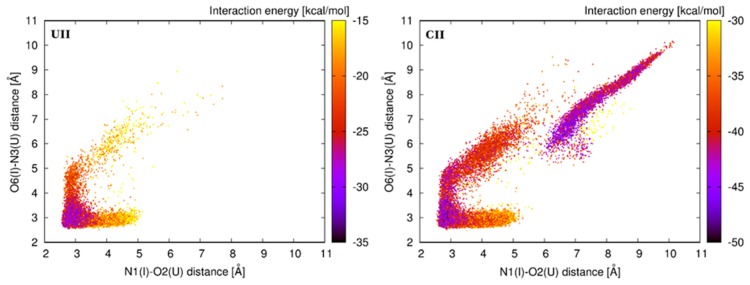

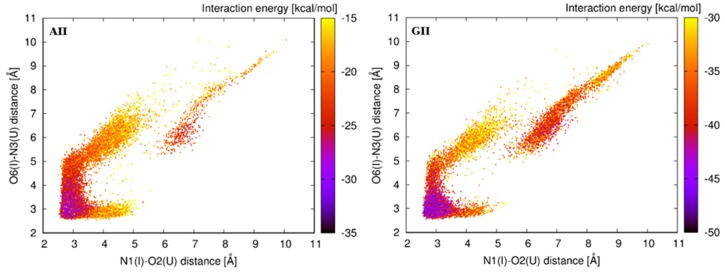
Distribution of the total interaction energy between the first I–U base pair and its 5′ base pair neighbor in simulations of the tandem I–U/I–U systems. Color scale represents the interaction energy.

The total interaction energy of the base pair step consists of the stacking energy and H-bond energies for each participating base pair. Distribution of the total interaction energy is presented in Figure 8. It is not surprising that the lowest interaction energy is observed for the initial A-geometry. Alternative geometries of the first I–U base pair are usually less stable with higher interaction energies. The CII system represents the exception, where alternative E- and F-geometries are stable as the initial A-geometry. This observation can explain why the I–U base pair in the CII context can frequently adopt these alternative geometries.

### 3.7. Analysis of Single I–U and A–U Base Pairs in ABF Simulations via Base Pair Parameter Shear

In the section above, we described the geometry of the first I–U base pair in tandem I–U/I–U systems using base pair parameter shear and opening based on ABF simulations (Figure 5). These results were correlated with the observed I–U geometries from the unbiased MD simulations. Moreover, we analyzed single I–U base pair in UI, CI, GI, and AI systems, as well as the standard A–U base pair in different sequence contexts in the wt system. Global minimum for A–U pair is around 0° (Figure S6). Local minima around −4 Å and 4 Å are separated by large energetic barriers of 7–8 kcal/mol. This corresponds to the behavior in the standard simulations, where A–U pairs are stable and do not fluctuate. In the case of the single I–U pair, the global energetic minimum is about −2.4 Å (corresponding to shift into the major groove) and there is an apparent lowering of the barrier into the minor groove, where a local minimum exists around 3.5 Å in all systems.

### 3.8. Global Characteristics of I–U Double-Stranded RNA

To capture stability of RNA duplexes, we analyzed their energy of formation and entropy. The most stable duplex was wt and systems with the single I–U base pair (Table 2). On the contrary, RNA duplexes with four and six inosine base pairs, that is, UII_CII and UII_CII_AII systems, respectively, were least stable. In comparison with the wt system, their energy of formation was higher about 10 kcal/mol.

The UII_CII and UII_CII_AII systems also show higher entropy when compared with wt and systems with the single I–U base pair (Figure S1 and Table 2), differences among systems differing by one I–U or two I–U pairs are not apparent. This confirms the observation from the experiments that the I–U base pairs destabilize the canonical RNA duplex. Calculations of the total bend describing the curvature of the duplex revealed no difference between wt system and duplexes with the single or tandem I–U pairs (Table 2). Thus, the global geometry of the duplex was not disrupted by the presence of I–U pairs, in agreement with a previous experimental study [19].

## 4. Discussion

RNA molecules with modified bases play a role in various cell processes [45]. These modifications are changes to the chemical composition of nucleotides catalyzed post-transcriptionally by specific RNA modification enzymes and occur in functionally important sites [46]. Modified residues change properties of natural RNA molecules, but their precise effect is mostly not known. Here, we focused on the analysis of dsRNA duplexes with single and tandem I–U base pairs (Figure 1). Previous studies have shown that the I–U pairs destabilize RNA duplexes [17,21,47], but little is known about the role of sequence context and behavior of the tandem I–U pairs.

We employed standard and biased simulations. Detailed structural analysis revealed that presence of the I–U pair induces changes in some base pair parameters and also affects local bend (Figure 3 and Table S2). The single I–U pairs were basically stable in all sequence contexts. In tandem I–U/I–U systems, increased flexibility was observed for the first I–U pair depending on its neighboring 5′ base. In particular, the CII system exhibited the largest dynamics (six base pair geometries were detected, see Figure 4) while the UII system was the most rigid. Basic I–U base pair A-geometry and alternative substates (geometries B–F) were detected in most tandem systems (Table 1). The order of stability can be expressed as U>G>A>C, while the experimentally known preference for 5′ neighbor editing is U>A>C>G. If we avoid the GII triplet from the comparison, as GA is rarely edited because of clashes between residues (UA is ca. 40 times more frequently edited than GA with ADAR1), we can see correlation between the stability of triplets and frequency of editing, that is, sequences that are frequently edited exhibit stability in RNA duplexes, and vice versa.

Observations from the standard simulations are supported by the energetic profiles from ABF calculations, where we perturbed the I–U pair via parameter shear and opening (Figure 5). To better understand the behavior of the first I–U in tandem systems, we analyzed interaction energy consisting of the H-bonding energy between bases of the I–U pair and the stacking energy of corresponding base pair steps. We observed that the H-bonding energy of the first I–U base pair is lowest for the basic A-geometry and less stable in alternative substates. The stacking energy of the first I–U from I–U/I–U and adjacent 5′ base pair was very similar for all geometries in AII, GII, and UII systems. However, in the CII system, this stacking energy decreased in the alternative geometries when compared with the A-geometry. Therefore, A-, E-, and F-geometries have equivalent total interaction energy in CII, while for other tandem systems, the A-geometry is the most stable (Figure 8). It correlates with the increased dynamics of CII system when compared with AII, GII, and UII. In agreement with previous studies, we observed that stability of RNA duplexes decreases with increased number of I–U pairs in the duplex (Table 2). The CII system exhibited the highest entropy among tandem systems, which probably correlates with the increased I–U base pair flexibility.

The ability of the first I–U pair to adopt various geometries in different sequence contexts indicates its plasticity, which might be critical for interaction with proteins of the immune response [48]. We discovered that most frequently edited sequences UAA (UII system) have lower flexibility than the other sequences (AII, GII, CII). More likely, the UAA motifs in ALU’s RNA do not have to be sufficiently effective in preventing immune signaling. Because significant sequence variations exist in ALUs among humans [49], some individuals could more likely be associated with increased risk for disease phenotype [50].

## Figures and Tables

**Table 1:** Percentage population of various geometries of the first inosine–uracil (I–U) base pair in the tandem I–U/I–U base pairs.

System Name	The Population of the Substate (in %)					
	A	B	C	D	E	F
UII	90	6	3	1	0	0
CII	56	21	4	7	6	6
GII	84	2	4	4	5	1
AII	76	4	12	7	1	0

**Table 2:** Energy of formation of RNA duplexes, entropy contributions, and total bend.

System	MM-PBSA (kcal/mol)	Δ*S*_∞_ (cal/mol·K)	Total Bend (°)
wt	−116 ± 10	585.4 ± 0.9	20
UI	−116 ± 9	588.4 ± 1.3	19
AI_1_	−113 ± 9	585.1 ± 1.2	21
CI	−117 ± 9	586.0 ± 0.2	21
AI_2_	−116 ± 9	583.0 ± 0.3	21
GI	−116 ± 9	587.1 ± 1.5	21
AI_3_	−116 ± 9	585.1 ± 0.3	23
AI_4_	−117 ± 9	585.0 ± 0.6	20
UII	−111 ± 11	586.4 ± 0.7	21
CII	−113 ± 9	596.6 ± 0.8	22
GII	−114 ± 8	588.6 ± 0.2	22
AII	−115 ± 9	587.8 ± 0.6	22
UII_CII	−107 ± 10	602.8 ± 1.7	21
UII_CII_AII	−103 ± 11	593.6 ± 1.8	21

MM-PBSA: Molecular mechanics, Poisson–Boltzmann combined with surface area, ΔS**_∞_**: Vibrational entropic contributions extrapolated to infinite time, Standard errors represent error of the intercept calculated with the linear regression.

## Supplementary Materials

The following are available online at http://www.mdpi.com/2073-4425/9/7/324/s1. Figure S1: Entropy contributions calculated over time windows 100–200 ns, 100–300 ns, 100–400 ns, and 100–500 ns for wt and UII_CII_AII system. Figure S2: Distribution of various geometries of the I–U base pair observed in the single I–U systems as a result of correlation between N1(I)–O2(U) and O6(I)–N3(U) H-bond distances. Figure S3: RMS deviations along the production trajectories evaluated for the tandem I–U/I–U systems. Figure S4: Distribution of various geometries of the first I–U base pair observed in the I–U/I–U tandem systems as a result of correlation between N1(I)–O2(U) and O6(I)–N3(U) H-bond distances. Figure S5: Selected structures from molecular dynamics (MD) simulations. Figure S6: Energetic profiles of A–U and single I–U pairs based on in the ABF simulations. Table S1: Two-term coefficients describing editing frequencies by ADAR1 and ADAR2 based on experimental study. Table S2: Averaged local bend values calculated for twelve selected systems.
