# Supplementary material for: Role of Inosine–Uracil Base Pairs in the Canonical RNA Duplexes
Source: Genes (Basel). 2018 Jun 28;9(7):324. doi: 10.3390/genes9070324 (PMC6070904; doi:10.3390/genes9070324)
Supplement: Supplementary file 1 [file genes-09-00324-s001.pdf]

Supplementary Materials

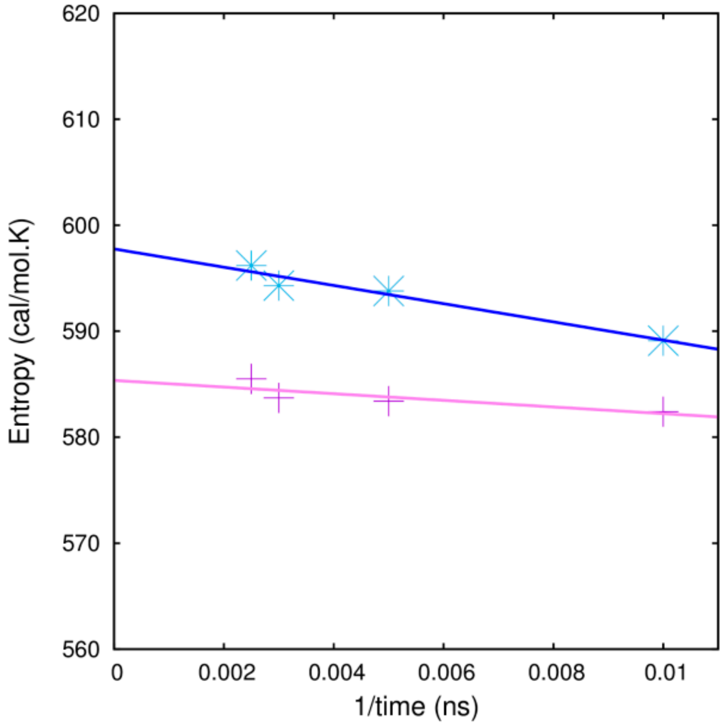

Figure S1. Entropy contributions calculated over time windows 100–200 ns, 100–300 ns, 100–400 ns, and 100–500 ns for the wild-type (wt) (purple crosses) and UII_CII_AII systems (blue asterisks).

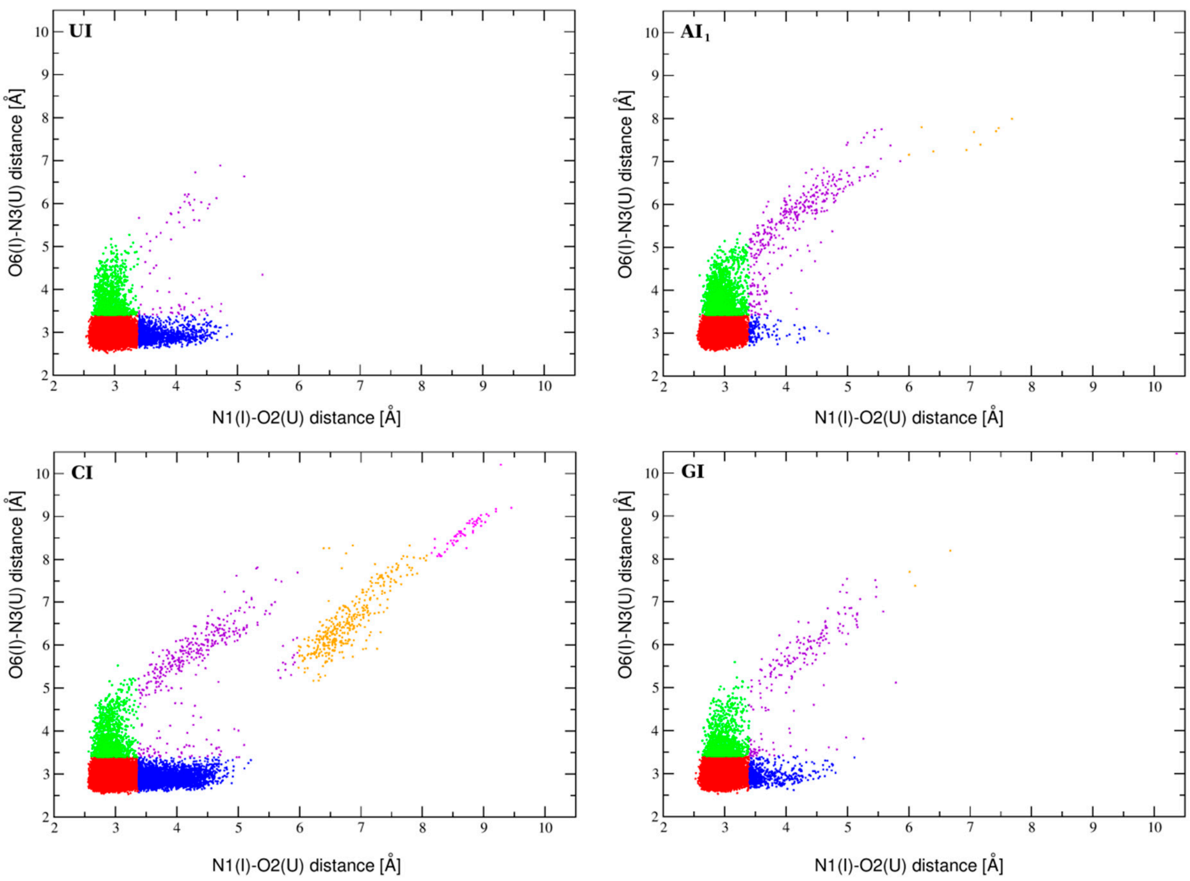

Figure S2. Distribution of various geometries of the inosine–uracil (I–U) base pair observed in the single I–U systems as a result of correlation between N1(I)–O2(U) and O6(I)–N3(U) H-bond distances. The AI_1 system was selected as a representative of the AI sequence motif. The initial geometry (in red) is predominantly populated (above 90%), while populations of B (in blue) and C (in green) geometries are ca. 1-5%, and D, E, and F geometries (in violet, orange, and magenta) do not exceed 1%. Colors of substates correspond to Figure 4 in the main text.

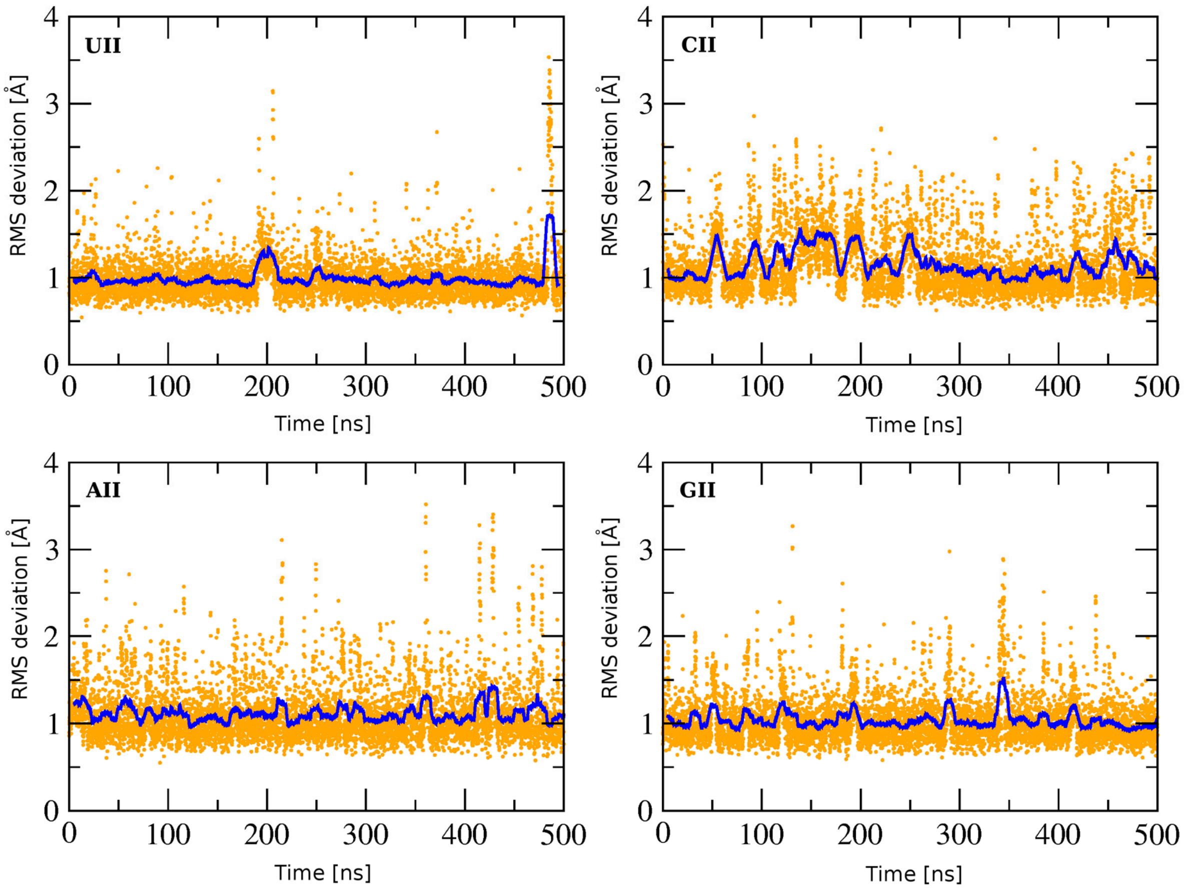

Figure S3. Root mean square (RMS) deviations along the production trajectories evaluated for the tandem I–U/I–U systems. RMSd was calculated for the I–U/I–U core supplemented by 5′ and 3′ base pair neighbors. The minimized structure with corrected I–U geometry was used as a reference.

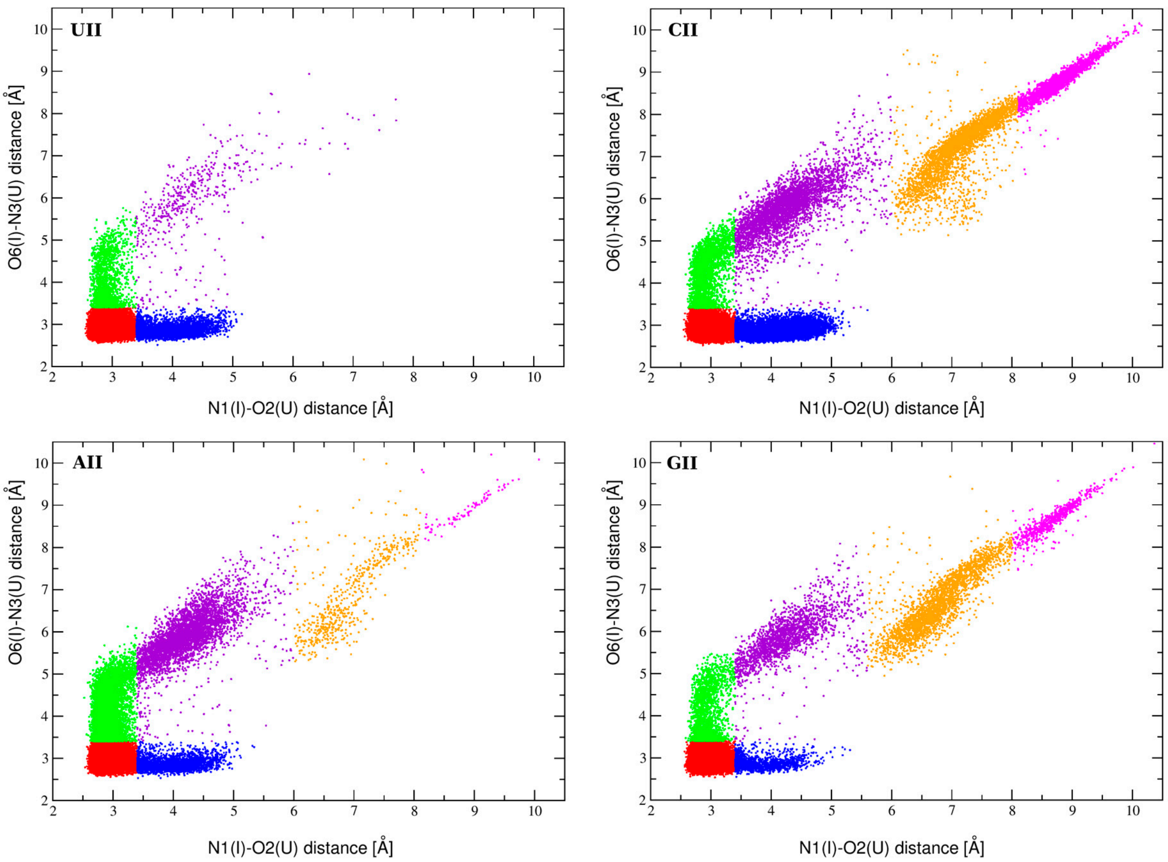

Figure S4. Distribution of various geometries of the first I–U base pair observed in the I–U/I–U tandem systems as a result of correlation between N1(I)–O2(U) and O6(I)–N3(U) H-bond distances. Substate populations are roughly distinguished by different colors, which corresponds to Figure 4 in the main text.

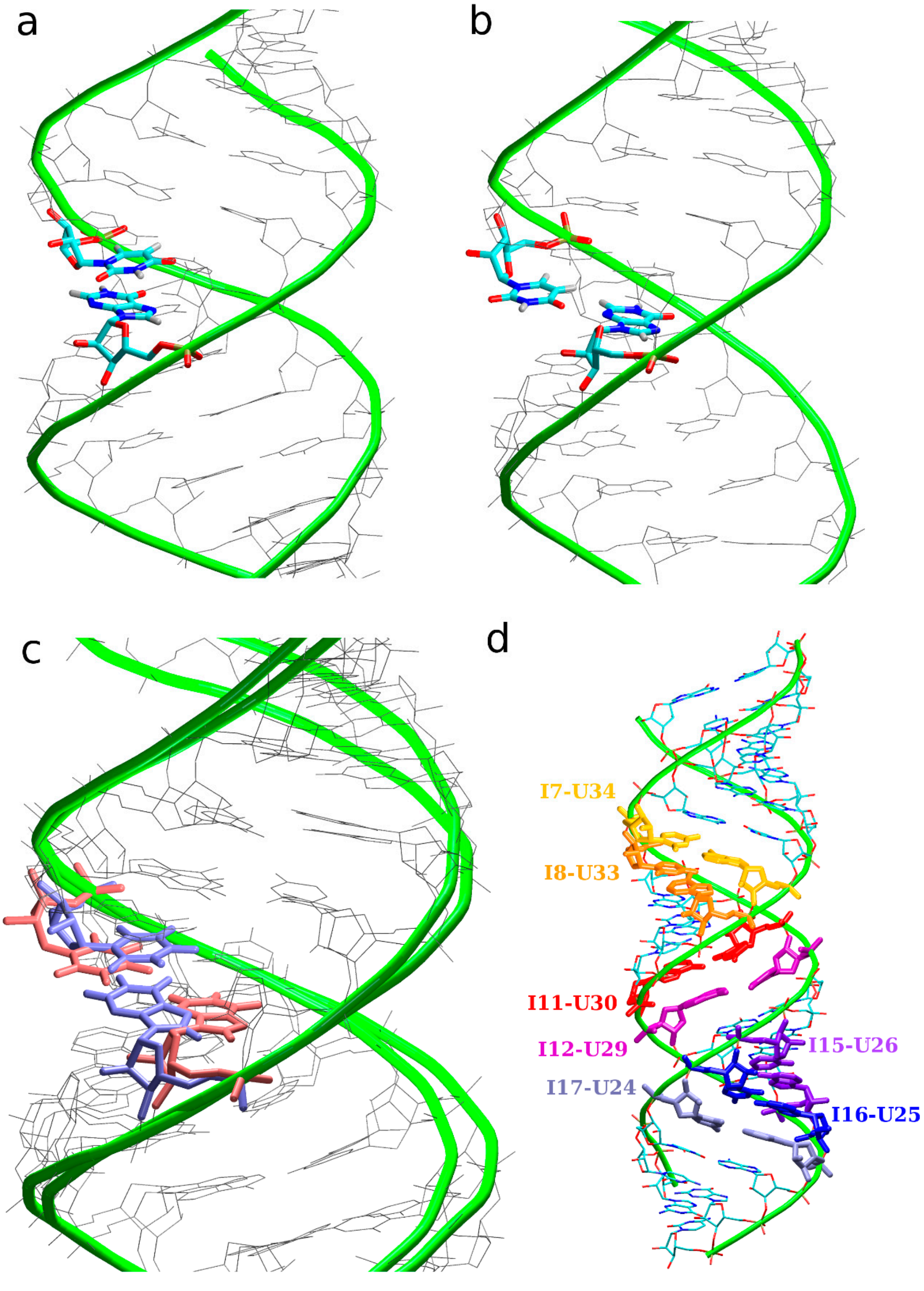

Figure S5. Selected structures from molecular dynamics (MD) simulations. (**a**) A snapshot of the UII system in the A-geometry (I7–U34 base pair is highlighted); (**b**) a snapshot of the CII system in the F-geometry (I11–U30 base pair is highlighted); (**c**) an overlay of A- and F-geometries in the CII system (the A-geometry in violet, the F-geometry in pink); and (**d**) distribution of I–U base pairs along the RNA duplex.

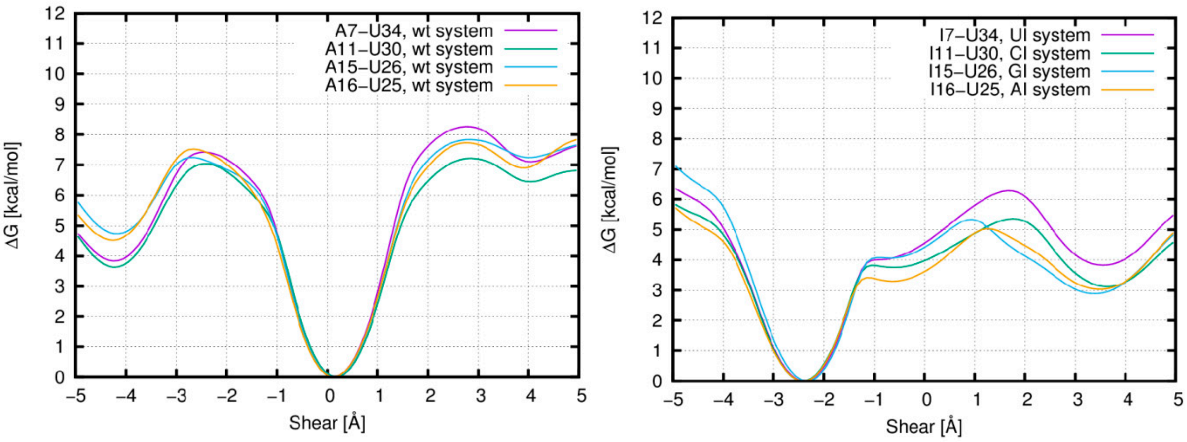

Figure S6. Free energy profiles of A–U and single I–U pairs based on adaptive biasing method (ABF) simulations.

Table S1. Two-term coefficients describing editing frequencies by adenosine deaminases acting on RNA (ADAR)1 and ADAR2 based on experimental study[1].

**base**	**coefficients for the first 5′ neighboring base (ADAR1)**	**coefficients for the first 3′ neighboring base (ADAR1)**	**coefficients for the first 5′ neighboring base (ADAR2)**	**coefficients for the first 3′ neighboring base (ADAR2)**
**A**	0.86	1.48	1.17	0.87
**C**	0.25	1.56	0.43	1.45
**G**	0.046	1.94	0.052	1.9
**U**	1.75	1	1.69	1

[1]Eggington et al., *Nat. Commun.* **2011**, *2*, 319, 10.1038/Ncomms1324

Table S2. Averaged local bend values calculated for twelve selected systems

**step**	**wt**	**UII**	**CII**	**GII**	**AII**	**UI**	**AI$_1$**	**CI**	**AI$_2$**	**GI**	**AI$_3$**	**AI$_4$**
1	6.7	6.7	6.9	6.8	6.8	6.8	6.7	6.8	6.8	6.7	6.8	6.8
2	14.4	15	14.9	14.7	14.6	15	14.5	14.8	14.7	14.7	14.6	14.8
3	11.7	12.8	11.8	11.7	11.6	12.3	12.1	11.8	11.8	12	11.7	11.8
4	9.7	11	9.6	9.5	9.5	10.1	10.3	9.4	9.6	9.8	9.6	9.6
5	11.5	11.5	11.2	11.3	11.3	10.7	11.7	11.1	11.1	11.4	11.2	11.4
6	14.7	15.8	14.5	14.7	14.5	15.5	15.2	14.3	14.2	14.5	14.5	14.7
7	9.5	**12.2**	10.1	9.5	9.4	9.8	11.4	9.9	9.5	9.5	9.4	9.4
8	7.7	7.6	8.1	7.6	7.6	7.7	7.9	7.9	7.7	7.6	7.6	7.6
9	10	11	9.4	9.9	10	9.5	11.8	9	10	10	10	10
10	11.9	11.9	10.6	11.7	11.8	11.2	12.7	11.4	10.9	11.9	11.8	11.8
11	8.6	8.3	**11.4**	9.1	8.5	8.8	8.4	8.7	**11.2**	8.9	8.6	8.5
12	9	9	8.2	9.8	9.5	9.2	8.9	9.1	8.5	9.4	9.5	9.1
13	10.4	10.3	10.3	10.7	11.2	10.4	10.2	9.7	10.8	10.4	11	10.7
14	10.7	10.8	10.4	11.2	9.7	10.6	10.7	10.5	10.5	11.7	9.9	11.1
15	10.7	10.8	10.5	**12.9**	11.4	10.6	10.7	10.7	10.6	11.6	12.1	10.1
16	11.3	11.3	11.3	12.9	**13.8**	11.1	11.1	11.3	11.1	12.2	11.9	13.2
17	10.4	10.3	10.6	11	10.9	10.2	10.3	10.3	10.6	9.7	11.1	10.3
18	10.3	10.3	10.5	9.6	11.3	10.5	10.4	10.3	10.4	10	9.9	12.3
19	6.5	6.5	6.5	6.5	6.2	6.5	6.6	6.5	6.5	6.6	6.4	6.2

Values larger than 2 Å when compared with the wt system are in bold.
